# Large-scale quantitative analysis of neurons via morphological structures by Fast Automatically Structural Tracing Algorithm (FAST)

**DOI:** 10.1186/1471-2202-16-S1-P228

**Published:** 2015-12-18

**Authors:** Nan-Yow Chen, Kuan-Peng Chen, Chi-Tin Shih, Guan-Wei He, Ting-Yuan Wang, Yu-Tai Ching, Ann-Shyn Chiang

**Affiliations:** 1 National Center for High-Performance Computing, Hsinchu 30076, Taiwan, Republic of China; 2 Department of Physics, Tunghai University, Taichung 40704, Taiwan, Republic of China; 3 Department of Computer Science, National Chiao Tung University, Hsinchu 30010, Taiwan, Republic of China; 4 Department of Life Science, National Tsing Hua University, Hsinchu 30013, Taiwan, Republic of China

## 

Quantitative analysis of neurons is a very important issue in neural science especially after numerous three-dimensional neural images in *Drosophila *brains were taken from confocal laser scanning microscope [1]. However, analyzing these messy data is mostly by human being with some semi-automatic software packages so far. Not only the task is very labor intensive but also the result is susceptible to errors and usually lacks objectivity. Therefore, fast and accurate analyzing tools are crucial and very desirable. Recently, we developed a computational algorithm, FAST (Fast Automatically Structural Tracing algorithm), which can trace neurons and get characteristic quantities of neuron fibers from their morphology in a very efficient way. These characteristic quantities (called SIs, Structural Indexes) are, for example, number of branch points, number of end points, cross section area of fibers, branch angle of fibers, distribution of fiber length, curvature of fibers, and innervation in neuropils, etc. After structural indexes of neuron fibers were obtained, isomap [2] and modularity [3] methods are applied to classify neurons without depending on human intervention. The isomap method can defined the similarity between neurons by geodesic paths in a high-dimensional manifold as well as the modularity method can find the best community structure of classification by optimization, i.e., to maximize the intra module connections as many as possible and to minimize the inter module connections as few as possible. With these tools, large-scale neural morphological structures, their annotations as well as quantified characteristics, and neural classifications can be facilely and reliably retrieved as useful data for computational neuroscience.

**Figure 1:**
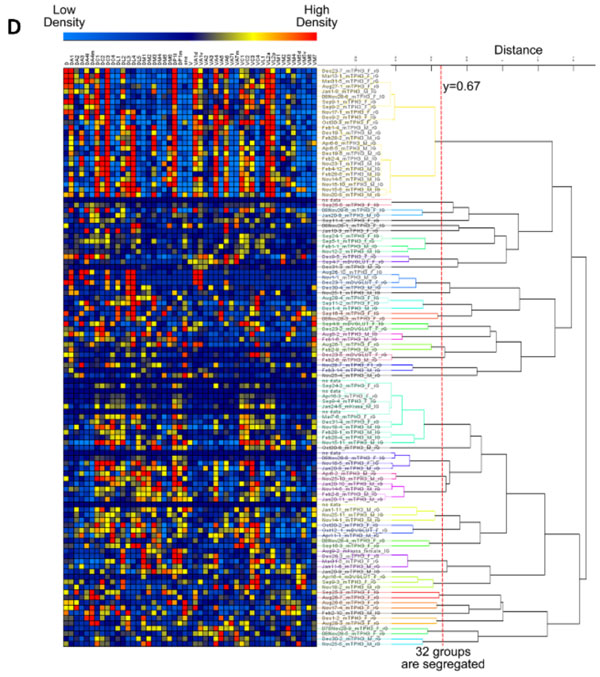
**A schematic diagram for innervation table and classification results of local neurons in olfactory system of *Drosophila***.
